# Bioactive Compounds from Red Microalgae with Therapeutic and Nutritional Value

**DOI:** 10.3390/microorganisms10112290

**Published:** 2022-11-18

**Authors:** Flora Tsvetanova, Dragomir Yankov

**Affiliations:** Institute of Chemical Engineering, Bulgarian Academy of Sciences, 1113 Sofia, Bulgaria

**Keywords:** red microalgae, valuable biochemicals, health benefits

## Abstract

Red microalgae represent a natural reservoir of beneficial substances with applications in different industrial sectors. They are rich in natural biomolecules known for their antihypertensive, antioxidant, antimicrobial, antiviral, anti-inflammatory, antitumor, and anticoagulant activities. Many red microalgae are a source of vitamins, minerals, photochemicals, polyunsaturated fatty acids, and a wide spectrum of polysaccharides. The content of their valuable compounds and their activities have turned red microalgae into cellular factories of special interest in food, nutraceutical, and pharmaceutical industries. Like all microalgae, the red ones are superior to traditional crops for the aims of biotechnology as they are renewable sources widely available in great quantities and are easy to culture. Moreover, some of the most studied red microalgae are generally recognized as safe. This review summarizes the valuable biochemicals from red microalgae and highlights their health and nutritional benefits.

## 1. General Overview of Red Microalgae

Microalgae are eukaryotic, photoautotrophic, single-cell organisms inhabiting diverse ecosystems, including terrestrial, aquatic, and airborne environments [1,2]. They utilize solar energy, water, and inorganic nutrients to reduce CO_2_ into complex organic compounds, and some of them are capable of surviving in extreme conditions [3]. One of the markers that divides the microalgae into different classes such as *Phaeophyceae*, *Chlorophyceae*, *Pyrrophyceae*, *Bacillariophyceae*, *Chrysophyceae*, and *Rhodophyceae* (or red microalgae) [4], is the pigment composition of the cells. The red microalgae constitute a major group, which still remains unappreciated and little exploited. This group includes eight genera, the most studied of which are *Porphyridium*, *Rhodella*, and *Rhodosorus*. They are morphologically the simplest of all the red algae. Their size varies between one and one hundred micrometers, depending on the species and stage of growth. In the cytoplasm, many organelles involved in microalgal metabolism are presented. The major part of red microalgae consists of spherical or ovoid unicells [5]. Their color is due to the phycobiliproteins in plastids which serve as light-harvesting pigment–protein complexes in photosynthesis. Among red microalgae, *Porhyridium* sp. are cultivated for the commercialization of high-value products, including omega-3 long-chain polyunsaturated fatty acids, polysaccharides, antioxidants, and pigments [6,7].

**Figure 1:**
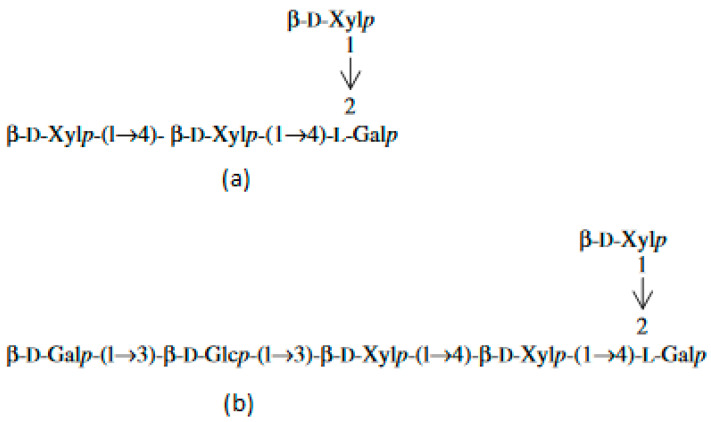
Oligosaccharides released from the extracellular polysaccharide of *Porphyridium* sp. after the uronic degradation with lithium in ethylenediamine. (**a**) oligosaccharide 1; (**b**) oligosaccharide 2.

Different from other microalgae, red ones do not contain microfibrillar cellulose. Their cell walls are encapsulated within a matrix of sulfated polysaccharides (PS). During the growth phase, the external part of the capsule dissolves into the medium, imparting an increase in viscosity. Another part remains membrane-bonded [8,9]. In the literature, these polymers are called EPS, meaning either extracellular polymeric substances, extracellular polysaccharides, or exopolysaccharides. The capsules are thinnest in the logarithmic growth phase and thickest in the stationary phase. The size of the capsule depends on the growth conditions. The thickness of the capsule is affected by the rate of production, degree of solubility, and cell surface area [4]. PS are composed of about ten different sugars, among which xylose, glucose, and galactose are present in higher quantities in different quantitative ratios. Rhamnose, ribose, arabinose, mannose, 4-O-methyl galactose, and 3-O-methyl pentose are present in minor quantities [10]. Red microalgae PS are negatively charged due to the presence of glucuronic acid and half-ester groups [11]. It has been found that the extracellular polysaccharide from *Porphyridium* sp. contains three neutral monosaccharides—xylose, glucose, and galactose, and one uronic acid—glucuronic acid. The uronic degradation with lithium in ethylenediamine resulted in two different oligosaccharides (Figure 1a,b). The chemical determination revealed the presence of D-xylose, D-glucose, D-, and L-galactose [12]:

All the red microalgae show a remarkable adaptation capacity to strong acid media (pH = 0.5–3.5) and high temperatures (38–56 °C) [13,14]. Such extremophile characteristics allow red algae to survive in habitats which other organisms cannot tolerate. They are capable of persisting in different geothermal environments and sulfur springs, boiling mud pools, and hot acidic waters [15]. Moreover, red microalgae tolerate metals; thus, they can survive in rather toxic conditions [16].

Microalgae are an important part of the food and nutraceutical industries due to their rich protein content. The microalgal protein content is higher than that of some vegetable sources such as rice, wheat, and legumes but poorer than the protein content of animal sources (meat and milk) [17]. Protein hydrolyzation results in releasing amino acids required for the growth and regeneration of the body and the maintenance of good health conditions. The microalgal protein content is affected by a variety of factors such as light intensity and spectrum, temperature, pH, and the nutrition media content [18]. Microalgal biomass is incorporated as a supplement in a wide range of health-promoting products such as tablets, gel capsules, and liquids. It is usually added to foods as protein supplementation or as a colorant in the form of dried powder—in pasta, snack foods, noodles, biscuits, ice cream, candies, and beverages [19]. In addition to their nutritional value, microalgal proteins play the role of precursors for the synthesis of antibodies, enzymes, and cytokines. Moreover, they can be used as oral vaccines, intestinal bioactive agents, and complex antitumor agents [20]. Algal proteins have already been used as food additives, dietary supplements, products of pharmaceutical value, and cosmetics. The prospect of the global algal protein market is to reach USD 0.84 billion by 2023, from USD 0.6 billion in 2018 [21].

The aim of the present paper is to summarize the published information on bioactive compounds in red microalgae with therapeutic and nutritional value, as well as on cultivation conditions, bioreactor design, and extraction methods.

## 2. Red Microalgae with Potential to Be Used in Food and Health Industries

Among red microalgae, the *Porphyridium* sp. (*Rhodophyta*), mainly *Porphyridium cruentum*, are the most explored and industrially employed ones on the grounds that they produce valuable biochemicals such as extracellular polysaccharides, phycobiliproteins, and long-chain PUFAs [22]. *Porphyridium* sp. are classified as GRAS (generally recognized as safe) by the Food Drug Administration [23]. The analysis of the nutritional composition of *Porphyridium cruentum* demonstrated its content comprised 32.1% protein, 21.7% crude fibers and 29.54% amino acids, and minerals such as Ca, Mg, Zn, and K [24]. This species is very convenient to work with, as it does not require vitamin B for its growth, contrary to other microalgae [25]. Another *Porphyridium* member is *P. sordidum.* Although it is a *Rhodophyta* member, it has a characteristic olive-green color. *P. sordidum* was first registered as a producer of exopolysaccharides (EPS) by the group of Cabrera. Compared to the EPS from *P. purpureum,* the EPS from *P. sordidum* show a higher sulfate content [26].

*Galdieria sulphuraria* is another red microalgae representative with great potential for biotechnological exploitation. It is characterized by a high daily productivity, accumulating biomass at a concentration of about 100 g/L dry weight. This red microalga is reported to utilize more than 27 different types of sugars and polyols and to tolerate glucose and fructose up to 166 g/L, salt concentration up to 1–2 M, and pH below 1 [27,28]. In addition, its cultivation under highly acidic conditions (pH 1.5–2.0) eliminates the possibility of bacterial contamination which is the main drawback in large-scale microalgae cultivation. The biomass is rich in proteins (26–32%) and polysaccharides (63–69%) and poor in lipids. Regarding micronutrients, *Galdieria sulphuraria* accumulates vitamins from group B, with beta-carotene as the main carotenoid, and phycobiliproteins [29]. It can be cultured heterotrophically which allows the cultivation to be conducted in cheap fermenters rather than high-cost photobioreactors [30]. High concentrations of phycocyanin are accumulated in the heterotrophic mode of culturing. Phycocyanin has found application in medical diagnostics as a fluorescent marker and a nontoxic coloring agent in the food and cosmetic industries [31].

The accumulated proteins are closely associated with the polysaccharide components and are, therefore, not digestible. As a whole, *Galdieria sulphuraria* possesses the potential to be incorporated in the human diet due to its protein-rich content and the presence of nondigestible dietary fibers. The nondigestible fibers play a very important role in human health as they can reach the colon and turn into nutrients for the beneficial lactic acid bacteria, thus improving metabolism and the immune system. In addition, the biomass is suitable to be implemented in functional foods, thanks to its low lipid content and the absence of green color [29].

*Dixoniella grisea (Rhodella reticulata)* is a little explored red microalga but is regarded as a potential source for lubricant production as it synthesizes a highly viscous polymer constituted of polysaccharides and proteins. These features would contribute a lot to the medical, cosmetic, and nutraceutical sectors, as conventional lubricants are toxic [32].

It was established that *Dixoniella grisea* possesses all of the four enzymes which take part in the mannitol cycle. In microalgae, the mannitol cycle guarantees the rapid accumulation or degradation of mannitol in cells as a response to salinity changes in the habitat. Mannitol is a type of sugar alcohol with applications in food, pharmaceutical, and medicinal products as a sweetener [33]. It has low molecular weight and caloric value and does not induce a glycemic response which poses an important factor for those with diabetes. As the consumption of processed sugars with a high caloric content is directly connected to obesity, diabetes, and other cardiovascular diseases, mannitol is one suitable substitute [34].

Table 1 summarizes the content of valuable substances in different species of red microalgae.

Clearly, red microalgae possess a great but not fully exploited potential to be used in food and health industries. A key objective of future research must be extending the number of industrially applicable red microalgae and broadening their produced chemicals with possible beneficial use.

## 3. Valuable Biochemicals with Therapeutic and Nutritional Potential Produced by Red Microalgae

Red microalgae are of significant biotechnological importance as they are a rich source of biomolecules with health and nutritional value. Polysaccharides, pigments, polyunsaturated fatty acids, and microelements are the highlighted beneficial compounds from red microalgae.

### 3.1. Polysaccharides

Thanks to their physical and chemical properties such as a high viscosity, high molecular weight, monosugar content, flexibility of the macromolecular chain, and level of sulfation, the exopolysaccharides of red microalgae have gained significant attention in recent decades [44]. The functions of PS are in accordance with the organism necessities in the environment it inhabits. For example, in the case of the red microalga *Porphyridium* sp. isolated from marine sand where the climate conditions are fluctuating, and illumination is strong, sulfated PS provide the required moisture [45]. Sulfated PS also act as a free radical scavenger which protects the cells from high solar irradiation [46]. It was also supposed that PS play the role of a buffer layer, preventing exposure from extreme pH values and temperatures [10]. PS are relatively stable in a wide range of temperatures (30–160 °C), pH (2–9), and salinity. PS can also provide a barrier against bacteria, viruses, and fungi [9].

Another important feature of the red microalgal PS for their industrial value is their dynamic fluid behavior which means that high solution viscosity is reached at a relatively low concentration of PS. This peculiarity lends microalgal PS similar qualities to those of industrial PS (for example, xanthan gum). In addition, microalgal solutions are stable at high temperatures, pH, and ionic strengths [9]. The EPS from *Porphyridium* are sustainable under environmental changes and against hyaluronidase-degrading enzymes. These qualities make them suitable to be employed as biolubricants. Liberman and co-workers found that EPS and their acid-hydrolysate fractions exhibit even higher antioxidant activity than carrageenan. They achieved ~70 and ~35% inhibition against *Escherichia coli* and *Bacillus subtilis*, respectively, with a 0.1% *w/v* of the EPS solution [47].

The biological activities of EPS are promoted by the presence of uronic acid and other bonded chemicals such as trace metals and proteins [48,49]. Comparing the physico-chemical characteristics of the polysaccharides of *Dixoniella grisea* and *Porphyridium aerugineum*, the group of Liberman concluded that the polysaccharides from brackish and fresh water species, such as *Dixoniella grisea*, are characterized by a smaller number of charged groups but demonstrate higher viscosity in comparison with *Porphyridium* sp. [48].

The typical sulfated structure of PS is responsible for various biological activities such as immunomodulatory, anti-inflammatory, hypocholesterolemic, antimicrobial, antiviral [50], antioxidant [51], and antihyperglycemic [52]. The sulfate content ranges between 1 and 4% (*w/w*) [53]. According to the bibliographic study, two families of PS are characteristic of red microalgae—the intracellular storage PS (IPS) and the extracellular structural PS (EPS). The basic IPS of microalgae are starch and starch-like PS. The IPS of red microalgae are floridean starch (floridean glycogen), an α–polyglucan structure similar to starch, lacking amylose [54]. On the other hand, some microalgae are known to contain amylose—*P. aerugineum*, *P. purpureum*, *P. sordidum*, *Rhodosorus marinus*, *Rhodella violaceae*, *Flintiella sanguinaria* [55]. Floridean starch is a storage polysaccharide present in the cellular component (cytosol) [56]. Sulfated PS are normally located as intracellular-bonded to the cytoplasmic membrane, as well as extracellular (exopolysaccharides) [8]. The EPS of *Porphyridium* are non-toxic, and their main sugar content includes xylose, galactose, and glucose, as well as glucuronic acid and sulfate groups [9]. The exopolysaccharides from *P. marinum* were reported to exhibit antimicrobial and anticancer activities. With the addition of only 31.3 μg/mL, the biofilm formation of *Candida albicans* was reduced by about 90%. The viability of the breast cancer cells was reduced by 55% [57].

Recently, a group of scientists assumed that the sulfated PS from *Porphyridium* could be used as a coating material on sanitary materials for COVID-19 prevention on the basis of their proven activity against respiratory viruses from the coronavirus family [58]. Huang and co-workers (2001) established that microalgal PS exhibit anti-hepatitis B activity [59]. Moreover, all of the red microalgal polysaccharide extracts demonstrate strong activity against the *V. stomatitis* virus, and this activity is even higher than the activity of all of the chemicals tested so far [60]. Huleihel and collaborators established that sulfur-containing polysaccharides in *Porphyridium* showed enhanced antiviral activity by blocking the adsorption of virions against HSV-1 and HSV-2 [61].

In recent years, lots of studies have demonstrated that microalgal PS can enhance antioxidant enzyme activity, eliminate free radicals, and inhibit lipid peroxidation [61]. Antihyperglycemic activity is important for diabetes therapy. This is a condition occurring in the case of enhanced blood glucose concentrations (hyperglycemia) and decreased insulin secretion [38]. Conventional drugs often lead to intestinal disorders. For that reason, the antihyperglycemic activity of external PS is one possible opportunity for the prevention of these undesirable effects. In a recent study, the antihyperglycemic effect of *P. cruentum*’s PS was demonstrated in vivo [52]. The antihyperlipidemic activities of red microalgae were proved by Dvir and colleagues in experiments with rats. The rats’ diet was supplemented with *Porphyridium* biomass, which resulted in a reduction in the levels of cholesterol, triglycerides, and very low-density lipoproteins and improved the hepatic cholesterol levels. These beneficial effects were observed due to the presence of dietary fibers and PS in *Porphyridium* [49].

Carrageenans, which belong to the group of sulfated PS, are known to have antiviral activity. Periera (2018) stated that carrageenans selectively inhibit the binding of many enveloped and nonenveloped viruses [62]. In a study by Grassauer and Grassauer (2011), they established that 400 µg/mL of carrageenan from red algae resulted in the inhibition of cell death caused by the coronavirus infection. They found out that if the cells were pretreated with carrageenans, they were protected against infection with coronavirus as well [63].

All of the above-listed features attach high value to red microalgae PS for their employment in pharmaceutic, cosmetic, and nutritional fields. PS from the red microalga *Porphyridium* are incorporated into nutraceutical products with antioxidant activity produced by the Solazyme company. This formula contributes to the reduction in inflammation and oxidative stress in mammalian tissues [64]. Becker and co-workers (2007) pointed out that the nutritional potential of red microalgae biomass is comparable to that of vegetables. Their advantage is the absence of rigid cell walls, thus making their proteins more accessible [35].

Microalgal PS have the potential to be added to cosmetic products, as they implement the function of strengthening the skin barrier and hydrating agent. A scientific group examined the sulfated PS of *P. cruentum* for their capacity to be incorporated in cosmetic and pharmaceutical products for the skin. They tested their activity on three enzymes with features contributing to good skin conditions. The results obtained confirm that PS are capable of reducing the activity of hyaluronidase and elastase [65]. This activity was patented by the Solazyme company in 2014 [66].

The three main activities of PS are patented—the stimulation of collagen, elastane synthesis, and collagenase inhibition. Another patent of PS in the cosmetic field concerns the improvement of the barrier function and/or skin hydration. This is the patent of the L’Oréal Company [67] and refers to a mixture of PS of the *Porphyridium* sp., sulfated PS from marine bacteria, and ulvan, associated with D-glucosidase. L’Oréal has patented sulfated PS for their antidandruff activity [68]. A patent from 2009 comprises red microalgae PS and heavy metals [69]. The deficiency of metals has raised a problem as more and more people suffer from them. PS, such as hyaluronate, can be used as a chelate in pharmaceutical products carrying the deficient metal [70]. This is the ground of Arad’s patent US20110070159A1 [69]. According to another patent of the Arad’s group (WO1997000689A1), the PS from red microalgae protect against virus infections, especially from the *Varicella Zoster* virus [71]. The company Frutarom in Israel specializes in the cultivation of *Porphyridium* sp. for the production of sulfated PS [72]. Greensea (Méze, France) and AlgoSolis (GEPEA, Université de Nantes, CNRS, France) commercially cultivate *Porphyridium cruentum* [58]. The exopolysaccharide of *Porphyridium cruentum* is claimed to be able to replace carrageenans in many applications [5]. Table 2 summarizes the industrial applications of the valuable compounds produced by *Porphyridium* and the companies processing them.

As a whole, the synthesis of exopolysaccharides is induced by nutrient limitation. The decrease in nitrogen leads to a stop in the microalgal growth and the beginning of the stationary phase. However, the microalgae proceed photosynthesizing and carbon fixation. Carbon is employed in the forming of energy reserves such as starch and lipids or is excreted in the form of exopolysaccharides [73].

### 3.2. Pigments

#### 3.2.1. Phycobiliproteins

**Figure 2:**
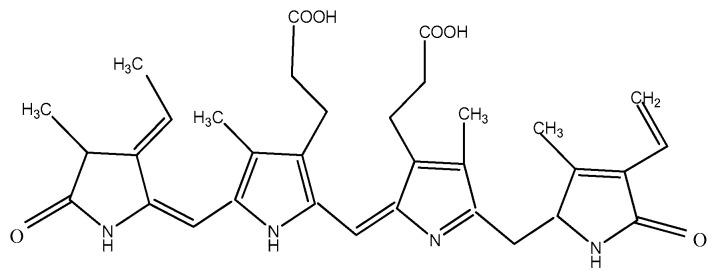
Structure of phycoerythrin.

**Figure 3:**
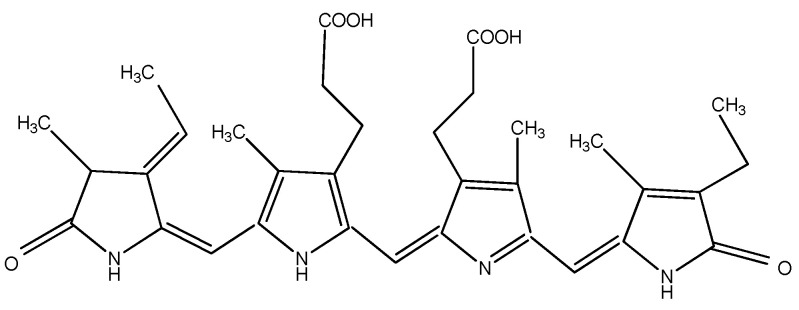
Structure of phycocyanin.

**Figure 4:**
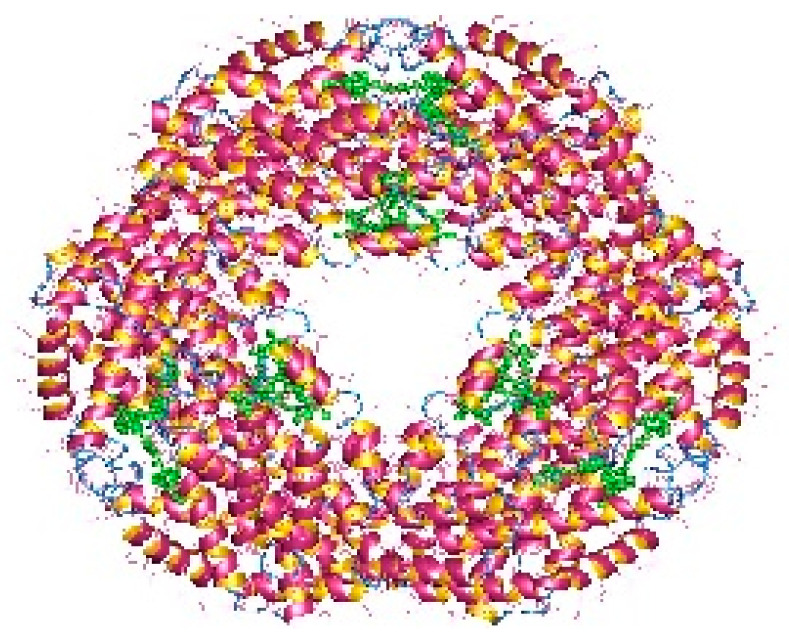
Allophycocyanin dodecamer (available online on https://en.wikipedia.org/wiki/Allophycocyanin, accessed on 14 November 2022).

The pigments documented to be produced by red microalgae are phycobiliproteins, chlorophyll, and carotenoids. Phycobiliproteins (PB) are a class of water-soluble colored proteins, located in phycobilisomes on the outer surface of the thylakoid membrane and are typical for three types of algae, featuring *Rhodophyta*, *Cyanophyta*, and *Cryptophyta*. They absorb light in the spectrum range of 450–650 nm [74]. Among phycobiliproteins, phycoerythrin (PE), phycocyanin (PC) and allophycocyanin are found in the *Rhodophyta* genus [75]. Their structures are presented in Figure 2, Figure 3 and Figure 4. Phycocyanin and allophycocyanin are approved as food colorants by the European Food Safety Authority. In *Porphyridium* sp., the main pigment is phycoerythrin (PE), constituting about 60–80% of the total soluble protein [76]. The pink/red color of *Porphyridium* is due to the content of phycoerythrin. The commercial PE used as a fluorescent dye is mainly obtained from large red algae but in low content (<0.1% of dry weight). The purification process is complicated and expensive [77]. In contrast, *Porphyridium* contains a high concentration of PE (about 8% of dry weight) and can be cultivated on a large scale [78]. PE demonstrates antioxidant, anti-inflammatory, and hepatoprotective properties [79]. The health impact of the antioxidant properties of phycobiliproteins is in reducing the rate of diseases such as cancer, diabetes, inflammation, and neurodegenerative disorders [6,61]. Richa et al. (2011) reported the potential for the commercial application of PB in biomedicine as antioxidant, anti-inflammatory, neuroprotective, hepatoprotective agents, and in fluorescence-based assays [80]. The effect of B-phycoerythrin (B-PE) from *P. cruentum* on the proliferation of Graffy myeloid tumor cells was studied in vitro. The results demonstrated an approximate 50 and 63% suppression of cellular growth when 50 and 100 µg of B-phycoerythrin, respectively, were added [81]. PE extracted from *Porphyra haitanensis* was shown to exhibit anticancer activities [82].

**Table 2:** Industrial application of bioactive compounds produced by red microalgae.

Species	Beneficial Substance	Application	Company/Product	Ref.
*Porphyridium* sp.	Pigments	Medical diagnostics, molecular biology	Greensea (Mèze, France)	[5]
	Sulfated polysaccharides	Oxidative cell protection, immune photo-protection, anti-inflammatory, anti-irritation	Frutarom (Haifa, Israel)	[5]
	Living phytoplankton	Aquaculture	Greensea (Mèze, France)	[5]
	Oligosaccharides derived from EPS	Vascular maintenance, heavy leg syndrome relief, rosacea and redness inhibition	Silidine^®^ by Greentech, St Beauzire, France	[39]
	sPS (sPS from marine bacteria and ulvan, associated with C-glycoside)	Improving the barrier function of skin, hydration	L’Oréal (Clichy, France)	[67]
*P. cruentum*	Sulfated Polysaccharides	Cellular regeneration, moisturizing agent (cosmetics)	AlgoSource (Saint-Nazaire, France)	[5]
		Antioxidant, anti-inflammatory, antimicrobial agent	Micoperi Blue Growth (Ortona, Italy)	[5]
		Sun-shielding, anti-inflammatory, antiaging	Asta Technologies (Haryana, India)	[5]
		Skin care	Solazyme (South San Francisco, CA, USA)	[5]
	Oligosaccharide	Vasoconstriction of blood vessels	Greensea (Mèze, France)	[5]
	B-phycoerythrin	Medical diagnostics, molecular biology, fluorescence techniques	Phyco-Biotech (Montpellier, France)	[5]
	Phycobiliproteins	Medical diagnostics	Phyco-Biotech (Montpellier, France)	[5]
	Biomass	Aquaculture	Isua^®^ Biotechnologie & Compagnie (Saint Just, France)	[5]
*P. purpureum* *P. cruentum*	Exopolysaccharides	Antioxidant activity	Alguard^®^ (Haifa, Israel)	[5]
*P. purpureum*	Phycoerythrin and EPS	Antiaging and sun care	Renouvellance^®^ (Microphyt, France)	[39]
	EPS	Melanin synthesis increase, enhances skin moisture and softness	Epsiline^®^ (St Beauzire, France)	[39]
	EPS	Skin hydration	Hydrintense^®^ (Vernier, Switzerland)	[39]
	Nondefined	Skin repair and hydration	algoVita (Tunis, Tunisia)	[39]
*Rhodella*	Concentrated biomass	Improves skin condition	Detoxondria, CODIF technologie naturelle (Saint-Malo, France)	[39]
*Rhodella violacea*	Complete extract	Skin hydration	Rosacea, CODIF technologie naturelle (Saint-Malo, France)	[39]
*Cyanidium* *caldarium*	Nondefined	Reduces the signs of skin aging	TEGO^®^ Stemlastin, (Essen, Germany)	[39]
*Rhodosorus* *marinus*	Nondefined	Soothes the nervous system	Mariliance, Givaudan (Vernier, Switzerland)	[39]

As natural pigments, phycobiliproteins are normally added to foods, cosmetics, and edible dyes. B-PE has become commercially valuable as a coloring agent. As it is not toxic, it is ready to be added to foods, drinks, and cosmetics [83]. The pigments synthesized by *Rhodophyta* are applicable in the pharmaceutical field for their fluorescence. PB are employed as fluorescent agents in medical diagnostics, immunochemistry, and bioengineering research [84]. They are shown to be good substitutes for radioimmunological tracers, with the same sensitivity of detection. Among PB produced by red microalgae, B-PE from *Porphyridium* is commercially available as a fluorescent agent for flow cytometry and immunofluorescent staining (by Invitrogen, Waltham, Massachusetts, USA; Colombia Bio-sciences, Maryland, USA; AnaSpec, Fremont, CA, USA) [85].

Phycocyanin (PC) is the major pigment in *Galdieria sulphuraria*. In general, other microalgae produce PC in minor concentrations which means that its commercial production is unprofitable. In contrast, *Galdieria,* as mentioned above, accumulates an enormous quantity of biomass of up to 100 g/L dry weight [27]; therefore, higher concentrations of PC are reached, even in a dark process [86]. PC concentrations produced by *Galdieria sulphuraria* are even higher than those produced by *Arthrospira (Spirulina) platensis* which is employed for commercial PC production and is dependent on the presence of light [86]. Phycocyanin and allophycocyanin find applications in various fields of industry: the food/feed market, pharmaceuticals, nutraceuticals, and cosmetics. In Japan, the company “Dainippon Ink and Chemicals Ink” imparts natural edible blue dye containing about 17% of phycocyanin. There are lots of Japanese patents concerning food coloring [4]. The market for 2025 is scheduled to reach USD 19.0 million for phycocyanin and 6.3 million for phycoerythrin [87].

#### 3.2.2. Chlorophyll and Carotenoids

**Figure 5:**
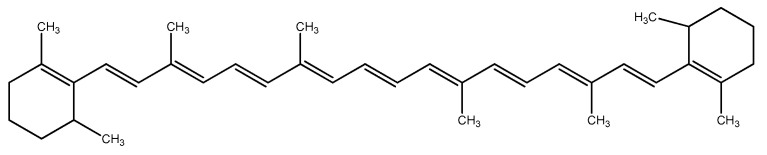
Structure of beta-carotene.

Chlorophyll *a* exists in all photosynthetic organisms. Chlorophyll *c* and *d* are registered to be synthesized by red microalgae from the *Rhodophyta* genus, as well as other pigments such as R—phycocyanin and α/β—carotenes [88]. Carotenoids are of special interest as they offer protection from solar radiation. The basic carotenoids in *Porphyridium* sp. are β-carotene (2.6% of the total carotenoid content) and zeaxanthin (97.4% of the total carotenoid content) [36], a small amount of fucoxanthin, violaxanthin, diadinoxanthin, and lutein [89]. Carotenoids are a common supplement as a source of vitamin A precursor in foods and as natural colorants in chewing gums [90]. They possess antioxidant activity, therefore, preventing diseases and protecting the skin [41]. Carotenoids have a definite capacity for degenerative disease prevention (macular degeneration and eye cataracts) as well [91]. Owing to the antioxidant properties of β-carotene (Figure 5), it was proposed for the prevention of cancer and chronic illnesses, for neurodegenerative diseases as a potential life-extender, and as an ulcer, heart attack, and coronary artery disease inhibitor. It also effectively controls blood cholesterol [18,92,93,94,95].

For zeaxanthin from *Porphyridium purpureum*, it was established that it induces apoptosis in human melanoma cells which suggests a capacity for adjuvant therapy [89].

### 3.3. Polyunsaturated Fatty Acids

**Figure 6:**
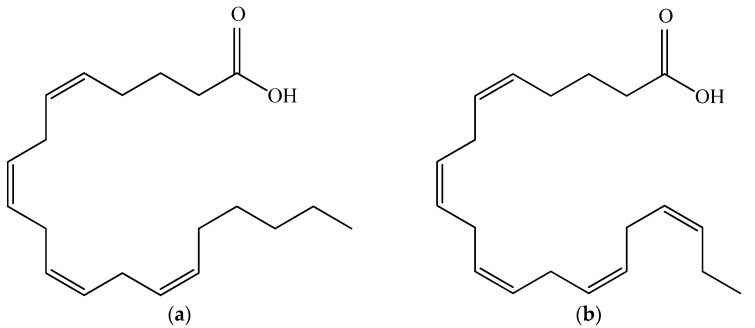
Structural formulas of arachidonic acid (**a**) and eicosapentaenoic acid (**b**).

Polyunsaturated fatty acids (PUFAs) are associated with very important physiological functions in human organisms. However, the human body is not capable of producing them. The common sources of fatty acids—marine fish oil and animal tissues—are diminishing [96]. *Porphyridium* is reported to synthesize a wide range of PUFAs, including palmitic, palmitoleic, stearic, oleic, linoleic, arachidonic (ARA) and eicosapentaenoic acid (EPA), as ARA and EPA comprise more than 40% of the total fatty acids. Their structures are shown in Figure 6. *P. purpureum* is claimed to be a promising accumulator of ARA, which is the most important PUFA, required for normal brain function, as an immune suppressant, a natural antifreeze, and an important intermediate for key physiological functions such as the metabolism of lipoproteins, blood flow, and white blood cell function [97]. Jiao and colleagues (2018) reported high levels of ARA in *Porphyridium* at 211.47 mg/L [98]. In the human body, EPA affects important systems and functions, including the cardiovascular system; it cleanses the arteries, treats atherosclerosis, diabetes, and high blood pressure, suppresses the inflammatory systems, and has a therapeutic effect on some types of cancer. Both EPA and ARA can be added as a supplement in functional foods, milk, and eggs. ARA, obtained from *Porphyridium*, is incorporated in infant formulas and in foods as a nutritional supplement [99]. The low cholesterol content and the fact that they are odorless make the microalgal PUFAs appropriate to be added to foods and nutraceuticals [100]. However, the production cost is not economically reasonable in comparison with other PUFA sources [4]. *P. cruentum* is biotechnologically employed for ARA, pigments (phycocyanin, phycoerythrin), and extracellular polysaccharide production.

The accumulation of lipids and the fatty acid composition are dependent on a variety of factors, including light intensity, temperature and nutrition in the culture medium, and the biomass productivity of the culture. For that reason, the light and temperature are controlled in order to achieve the maximal ratios of ARA and EPA [4].

### 3.4. Micronutrients

Red microalgae are known to produce a variety of health-boosting vitamins such as A, B1, B2, B6, B12, C, E, nicotinate, biotin, folic acid, and pantothenic acid [35]. The vitamin content of microalgae is of great importance for their implementation as food additives. *Porhyridium* is capable of accumulating large quantities of tocopherols (vitamin E). The tocopherols are lipid-soluble antioxidants that protect the membrane lipids from oxidative stress [101]. Vitamin E, extracted from *P. cruentum,* is a key factor in the prevention of many diseases such as atherosclerosis, heart disease, and multiple sclerosis [102,103]. Durmaz and colleagues reported the accumulation of 55.2 µg/g of dry weight α-tocopherol and 51.3 µg/g of dry weight γ-tocopherol [37]. *Galdieria sulfuraria* was also reported to synthesize vitamin E in different quantities, depending on the operating conditions—9 and 15 mg kg^−1^ of dry weight for autotrophic and heterotrophic culture, respectively. With respect to water-soluble vitamins, the same microalga was demonstrated in the heterotrophic growth accumulation of vitamin B2 (30 mg kg^−1^) and B3 (32 mg kg^−1^). In the autotrophic growth, the content of vitamin B3 was only 20 mg kg^−1^, and no vitamin B2 was produced [29].

The red microalgae produce a great variety of biochemicals with beneficial effects on human health. Considering the fast-growing human population, the importance of red microalgae as a source of biochemicals with nutritional and medicinal applications will increase to meet human needs.

## 4. Cultivation of Red Microalgae

Red microalgae are being cultivated in open ponds or in closed systems. Open ponds provide lower costs and simpler cultivation processes. However, they lack temperature and illumination control, and there is a risk of contamination by other microalgae, bacteria, or protozoa. In addition, a high loss of water as a result of evaporation and CO_2_ diffusion in the atmosphere is observed [104]. For minimizing some of the drawbacks, cultivation in closed systems is often preferred. These constructions show the better performance and control of pH, temperature, and light intensity, and a much higher biomass productivity. On the other hand, closed systems require higher construction costs [105].

The bioreactor design for red microalgae cultivation does not differ from those for other microalgae. The types of photobioreactors, designed for microalgal cultivation are tubular, flat plate, and column. The tubular photobioreactors are usually built with glass or plastic tubes, and the culture is homogenized through an air pump. Some of the limitations are the fouling and pH variation. The construction of the tubular photobioreactors can be horizontal/serpentine, vertical, near horizontal, and inclined [106]. The flat plate reactor possesses a wide surface exposed to light and a higher cell productivity in comparison to the tubular one. The construction material is transparent for better light energy capturing. A disadvantage of this type of construction is the difficult temperature control. The column photobioreactors are characterized by easy operation, sterilization, and scaling-up. They offer a low consumption of energy, good homogenization with low shear stress, low photoinhibition and photo-oxidation, and a big surface area-to-volume ratio. The drawbacks include a small illumination area and the fact that the light intensity inside the bioreactor decreases with the scale-up [105]. Experiments aimed at improving results, performed in different types of bioreactors with *Porphyridium* (which is the most employed red microalgae genus), are presented in Table 3.

For achieving augmented biomass accumulation and valuable product yields, the operating conditions are of primary importance. One of these factors is the light regime. Extreme light intensity may provoke photo-oxidation, while low light intensity may inhibit growth [106]. The photon flux density (PFD) is in a close relationship with the cellular metabolism. Low PFD enhances protein synthesis. On the other hand, the content of extracellular PS boosts with the increase in PFD [107]. In addition to sunlight, artificial light is commonly used. There are reports concerning the effect of the different wavelengths on polysaccharide production. Blue light was reported to be an efficient instrument for the improved cell growth of *Porphyridium cruentum* and PS synthesis [108].

Temperature is another factor affecting the growth and accumulation of valuable products in red microalgae. *Porphyridium* are a mesophilic species, and the optimal temperature for their growth is around 20 °C. Above this value, their growth is inhibited [109]. The thermophilic *Galdieria sulphuraria* tolerates high temperatures of up to 56 °C. [12] Its thermophilic nature contributes to the prevention of bacterial contamination, especially in industrial conditions.

Temperature stress influences not only the growth but also the chemical composition of red microalgae. However, the temperature optimum is strain-dependent [5,109].

With respect to nutrients, the most important elements required for microalgal growth are carbon, nitrogen, phosphorus, and sulfur. Carbon is used mainly in the form of CO_2_. It is of basal importance for the microalgae as the majority of them are autotrophs. The direct sparging of CO_2_ into the culture could be an efficient method for augmented biomass and pH control. CO_2_ can be uptaken by the cells through active transport or passive diffusion [110]. Another option for carbon assimilation is in the form of sugars. A broad spectrum of sugar kinases is needed for metabolizing organic substrates. In addition to the glucose transporter, four putative sucrose transporters were reported for *Porphyridium purpureum.* Therefore, it is possible for that microalga to utilize disaccharides such as sucrose for heterotrophic growth [111]. *Galdieria sulphuraria* is capable of metabolizing more than 27 different sugars and polyols, featuring disaccharides, hexoses, pentoses, deoxysugars, hexitols, pentols, amino acids, and some organic acids [27].

Some microalgae are also capable of using HCO^−^_3_ as a carbon source, but preliminary conversion to CO_2_ or a transport system is required [112]. Nitrogen is a component in key enzymes, photosynthetic pigments, genetic materials, and other substances, necessary for microalgal growth. Although the most common form of nitrogen in seawater is nitrate, many microalgae preferably uptake ammonium. Therefore, all microalgae cells are supposed to use both forms of nitrogen. However, *Galdieria sulfuraria* is an exception as it can only metabolize ammonium [113]. In studies comparing the utilization of ammonium and nitrate, *P. cruentum* reached an improved growth rate and biochemical composition when ammonium was used as a nitrogen source. [114]. *Porphyridium* cells are also able to metabolize urea as a nitrogen supplement but only after a significant adaptation period [115].

In general, nitrogen starvation may lead to lipid and carbohydrate accumulation, but it would suppress the protein synthesis [116] and inhibit phycoerythrin production. Limited nitrogen is conducive to the dissolution of PS in the medium [117]. Phosphorus is a constituent in algal cellular nucleic acids, proteins, and phospholipids, and a key component in chlorophyll synthesis [118]. *Porphyridium* spp. use mainly H_2_PO_4_^−^ and HPO_4_^2−^ as a source of phosphorus. Su and collaborators (2016) concluded that there is an optimal phosphorus value, below or above which the growth of *Porphyridium* is not satisfactory [97]. Other nutrient compounds required by *Porphyridium* spp. are vitamins, especially from group B. B12 was reported to demonstrate the most promoting effect on *P. cruentum* growth [119].

**Table 3:** Cultivation of strains of *Porphyridium* in different types of bioreactors: conditions, improved results, and valuable product accumulation.

Strain	Nutrient Media, Conditions	Type of Photobioreactor	Product/Yield/ Result Achieved	Ref.
*Porphyridium* sp. UTEX 637	ASW *; irradiance of 150 μmol photon m^−2^ s^−1^; aeration with air with 1–3% CO_2_; 24 ± 3 °C; cultivation time: stationary phase of growth	1 L column Reactor	Enhanced antioxidant activity of PS	[51]
*Porphyridium* *cruentum*	ASW; blue light; PFD of 70 µE m^−2^ s^−1^; 25 °C; cultivation time: stationary phase of growth	Stirred airlift reactor	PS production yield of 0.95 g·L^−1^	[108]
*Porphyridium* *cruentum 2727* (strain UTEX 161)	Hemerick culture medium; 25 °C	10 L tubular air-lift reactor	6.0 mg/L EPS	[43]
*Porphyridium* *cruentum* *(strain* P.C-03)	Optimized OM Ι medium; PFD of 80 µE m^−2^ s^−1^; light–dark cycle of 18:6; 23–25 °C; cultivation time: 15 days	15 L flat plate photobioreactor	Max growth rate of 0.32 day^−1^; max cell density of 137.9 × 10^8^ L^−1^; PS production: 0.95 g·L^−1^	[120]
*Porphyridium* *cruentum 2727* Naegeli	Modified f/2 medium, final nitrate concentration of 8.82 × 10^−3^ M; final phosphate concentration of 3.62 × 10^−4^ M; PFD of 100 µmol m^−2^ s^−1^; 25 ± 1 °C	Attached cultivation column reactor	PS production of 42% dry weight	[121]
*Porphyridium* *marinum 2727* CCAP 1380/10	Pm medium; PFD of 360 μmol photons.m^−2^ s^−1^; 28 °C; cultivation time: 7 days after entering the stationary phase of growth	5 L cylindrical, radially illuminated photobioreactor	EPS concentration of 2.5 g·L^−1^; EPS productivity of 0.149 g·L^−1^·day^−1^	[122]
*Porphyridium* *cruentum*	Enriched ASW (2.8 gL^−1^ NaCl); 10.4 × 10^−3^ M KNO_3_ 5.5 × 10^−4^ M KH_2_PO_4_ light intensity of 96 μmol m^−2^ s^−1^); 18 °C	10 L tubular bioreactor	415.88 ± 17.95 μg g^−1^ of β-carotene; 1513.12 ± 61.78 μg g^−1^ of chlorophyll *a*; specific growth rate of 0.70 d^−1^	[123]

* ASW—artificial seawater.

Cultivation conditions might considerably influence the growth rate and quantity of the biochemicals produced. For example, nitrogen deficiency can result in a growth rate decrease, the accumulation of lipids, and EPS synthesis. Temperature changes can also affect the chemical production in red microalgae; lower temperatures (around 25 °C) favor the synthesis of pigments and polysaccharides, while higher temperatures are beneficial for the accumulation of proteins and fatty acids. Changes in the light regime (light intensity or light-to-dark period ratio) also alter the composition of produced chemicals. To some extent, the choice of the bioreactor design can also influence the ratio of the main groups of the produced chemicals.

## 5. Eco-Friendly Techniques for Extraction of Valuable Substances from Red Microalgae

To be utilized for the necessities of food and pharmaceutical industries, the valuable substances have to be extracted and purified. Bioactive molecules are sensitive to extraction techniques which include high temperatures and treatment with aggressive solvents. The purity and activity of the extracted compound depend on the extraction method.

The techniques for the extraction of valuable biochemicals are common for all microalgae. There are no special extraction techniques employed for the red microalgae only. Most techniques employ heating which harms PUFAs and PE. Therefore, these substances should be extracted before the PS. In general, PS are extracted using acid, alkaline, and water means. The basic extraction methods for PE use microwave-assisted extraction, in situ stirring extraction, and the freezing/thawing method (freezing at −20 °C for 2 h, followed by thawing at room temperature) [124]. As regards light and pH, PE requires mild conditions [125]. Lipids are usually extracted with ethanol, which is an economically viable polar organic solvent. However, there are no data about lipid extraction from *Porphyridium.* Heating accelerates lipid extraction but deteriorates the oxidation of the microalgal PUFAs [126]. The group of Li established a three-step fractional extraction mechanism for PE, lipids, and PS from *Porphyridium*, which employs cold water, 95% ethanol, and hot water. They achieved a total extraction rate of 78.5%, with reduced purity. The extracted PE showed a remarkable fluorescence activity. The crude lipid demonstrated a large number of membrane lipids. The extracted PS comprised mainly glucose, xylose, and galactose. Crude PE proved the higher antioxidant activity [38].

Recently, extraction methods denominated as “green” have gained rising popularity. These technologies aim at improving the extraction yields, quality, and purity of the bioactive natural compounds in a safe manner. They combine the utilization of GRAS (generally recognized as safe) solvents and reduced energy consumption [127,128]. Among these modern techniques are microwave extraction (MWE), ultrasound extraction (USE), supercritical fluid extraction (SFE), accelerated solvent extraction (ASE) (also called pressurized liquid/solvent extraction (PLE/PSE)), and subcritical solvent extraction [128]. The MW employs MWE radiation (300 MHz–300 GHz), which is transferred to the solution, provoking the disruption of the molecular bonds and dissolved ion migration. The solvent rebounds to the extraction of the bioactive compounds. The US method (frequency above 20 kHz) is based on cavitation. In this case, the increased mass transfer using eddy and internal diffusion mechanisms increases the extraction efficiency. SFE uses supercritical fluids (fluids at a temperature and pressure above their critical point as the extraction solvent) [129]. The advantage of this extraction method is the possibility to adjust the solvating power of the supercritical fluid using temperature and pressure manipulation, allowing the selective extraction of multiple compounds. The preferred solvent is usually CO_2_, as it is characterized by low toxicity, flammability and cost, and relatively high purity [130]. A group of scientists combined conventional extraction techniques (maceration and freeze/thawing) with green extraction techniques (MWE and USE) for the extraction of PE from *P. cruentum* and *P. purpureum.* They established that the USE method was the most effective method for both species [23].

The choice of appropriate extraction technique is of great importance because it directly influences the yield, quality, and price of the high-added value chemicals obtained from red microalgae.

## 6. Perspectives

Red microalgae are recognized as a potential source of various compounds with nutritive and therapeutic value. Despite the broad knowledge of the cultivation conditions, metabolite variety, and separation methods, there are some points that have to be improved towards turning the red microalgae into a real biorefinery, converting sunlight into numerous chemicals with high-added value. Some of them are listed below:

- Describing new species and strains with a better tolerance to environmental stress and an increased expression of valuable substances.
- Applying new effective, selective, and low-cost methods for the separation of the desired compounds.
- Developing new analytical and genetic tools for a better understanding of the chemical composition, biochemical pathways, and physiological processes in red microalgae.
- Closing the gap between laboratory and large-scale production, keeping a steady ratio between different classes of the produced chemicals.

## 7. Conclusions

Environment care and protection, along with human health and nutrition, are factors of primary importance. Therefore, green methods and technologies in every cluster of the industry are to be employed. Microalgae as a component of the human diet were well-documented over 2000 years ago in China. Nowadays, scientists have established that red microalgae represent green factories, implementing numerous valuable bioactive composites with an emphasized health impact, which make them an excellent perspective tool for the purposes of biotechnology. In addition, microalgae have the lowest carbon, water, and arable footprint of any other crop; therefore, they are characterized by long-term sustainability. In addition, compared to other terrestrial crops and animal foods, microalgae demonstrate very high productivity. The development of successful biotechnology, involving environmentally sound practices for red microalgae is one of the footsteps in the direction of improving nature and human maintenance.

## Figures and Tables

**Table 1:** Protein, polysaccharide, PUFA/lipid, and carotenoid content of red microalgae species.

Species	Protein Content (% Dry Matter)	PS Content (% Dry Matter)	PUFA/Lipid Content	Carotenoids	Ref.
*Porphyridium* *cruentum*	28–39	>50	43.7% of total fatty acids	19.11 ± 4.33 (mg g^−1^ extract) zeaxanthin; 43.15 ± 0.84 (mg g^−1^ extract) total carotenoids	[35,36,37]
*Porphyridium* *purpureum*	15.08	>50	40% of total fatty acids	396.7 ± 0.3 µg g^−1^ dry weight β-carotene; 586.3 ± 0.3 µg g^−1^ dry weight zeaxanthin	[38,39,40]
*Porphyridium* *aerugineum*	32	No data	5–9% of total fatty acids	0.4 ± 0.01 mg g^−1^ dry weight zeaxanthin; 0.4 ± 0.07 mg g^−1^ dry weight β-carotene	[41,42,43]
*Galdieria* *sulphuraria*	26–32	63–69	Poor	575 ± 123 mg kg^−1^ astaxanthin; 387 ± 112 mg kg^−1^ lutein	[29]
*Dixoniella* *grisea*	10% cellular and 7% released	56	4–5% of lipids	No phycobilisomes	[5,32]
